# Possible Role of the *WDR3* Gene on Genome Stability in Thyroid Cancer Patients

**DOI:** 10.1371/journal.pone.0044288

**Published:** 2012-09-26

**Authors:** Wilser Andrés García-Quispes, Susana Pastor, Pere Galofré, Josefina Biarnés, Joan Castell, Antonia Velázquez, Ricard Marcos

**Affiliations:** 1 Grup de Mutagènesi, Departament de Genètica i de Microbiologia, Universitat Autònoma de Barcelona, Bellaterra, Spain; 2 CIBER Epidemiología y Salud Pública, Instituto de Salud Carlos III, Madrid, Spain; 3 Servei de Medicina Nuclear, Hospitals Universitaris Vall d'Hebron, Barcelona, Spain; 4 Unitat d'Endocrinología, Hospital Josep Trueta, Girona, Spain; University of Navarra, Spain

## Abstract

The role of the *WDR3* gene on genomic instability has been evaluated in a group of 115 differentiated thyroid cancer (DTC) patients. Genomic instability has been measured according to the response of peripheral blood lymphocytes to ionizing radiation (0.5 Gy). The response has been measured with the micronucleus (MN) test evaluating the frequency of binucleated cells with MN (BNMN), both before and after the irradiation. No differences between genotypes, for the BNMN frequencies previous the irradiation, were observed. Nevertheless significant decreases in DNA damage after irradiation were observed in individuals carrying the variant alleles for each of the three genotyped SNPs: rs3754127 [−8.85 (−15.01 *to* −2.70), P<0.01]; rs3765501 [−8.98 (−15.61 *to* −2.36), P<0.01]; rs4658973 [−8.70 (−14.94 *to* −2.46), P<0.01]. These values correspond to those obtained assuming a dominant model. This study shows for the first time that *WDR3* can modulate genome stability.

## Introduction

Chromosome region 1p12 has been associated to different types of cancer, including differentiated thyroid carcinoma (DTC) [1], [2]. Previous studies conducted by our group in this region have found that the *WDR3* gene, mapped in this region, shows a significant association with DTC, suggesting its implication in the aetiology of thyroid cancer [3].

Little information exists on the role of *WDR3*, but it is known that it belongs to a family of eukaryotic genes carrying WD repeats [4]. WD repeats are minimally conserved regions of approximately 40 amino acids, typically bracketed by Gly-His and Trp-Asp, which may facilitate formation of heterotrimeric or multiprotein complexes. Since WD-repeat proteins do not exist in prokaryotic genomes, it is assumed that probably they arose in the immediate precursors of eukaryotes [5]. Proteins belonging to the WD repeat family are involved in a variety of cellular processes; including cell cycle progression, signal transduction, apoptosis, and gene regulation [6]. Regarding to the *WDR3* gene, it should be mentioned that it encodes a nuclear protein of 943 amino acids containing 10 WD repeats [4]. This gene is involved in cell cycle progression and signal transduction [7] and, although the specific function of *WDR3* is unknown, a role in ribosome biogenesis has recently been reported [8].

Since neither of the functions attributed to WDR3 can be specifically associated with thyroid cancer, we hypothesized that perhaps it is involved in more general mechanisms maintaining genomic stability. Consequently, variations in the *WDR3* function associated with the existence of genetic polymorphisms can generate genome instability that can result in an increased cancer risk. In this context, it must be indicated that ribosomal proteins have been reported to be also involved in maintaining genome integrity [9].

Although genomic instability is a complex parameter, a general characteristic of individuals showing diseases such as Fanconi's anemia or ataxia telangiectasia is a poor response against agents affecting the integrity of the genome, as it is the case of ionizing radiation (IR). Thus, radiosensitivity is considered a biomarker of genomic instability [10]. To determine if a candidate gene is involved in genomic stability it must be demonstrated that cells carrying different genetic polymorphisms are particularly sensitive in front of the IR action.

Here, we evaluated the relationship between three non-coding single nucleotide polymorphisms of the *WDR3* gene and the frequency of micronucleus, both spontaneous and after IR exposure. The study has been carried out in peripheral blood lymphocytes from a group of 115 DTC patients, and the levels of DNA damage have been evaluated by using the cytokinesis-block micronucleus assay (CBMN), because it is a well-established cytogenetic technique with many advantages over other cytogenetic approaches. In this context, it must be stressed that it has been well demonstrated that CBMN assay is very useful as a marker of spontaneous and induced DNA damage [11]–[13].

**Table 1:** Spontaneous and after irradiation BNMN frequencies, according to gender and histological DTC type.

Patients (n)	Spontaneous BNMN (mean ± SE)	*P* a	BNMN after irradiation (mean ± SE)	*P* b
Total (115)	6.87±0.55		31.35±1.51	
Female (93)	7.19±0.65	0.339	31.19±1.70	0.833
Male (22)	5.51±0.94		31. 01±3.11	
Papillary (99)	6.64±0.57	0.559	31.71±1.50	0.553
Follicular (16)	8.31±1.32		29.13±5.53	

a Wilcoxon signed rank test.

b Student's t-tests.

## Materials and Methods

### Ethic statement

The study was approved by the Ethic Committees from the Hospital Universitari Vall d'Hebron in Barcelona, and the Hospital Universitari Doctor Josep Trueta in Girona (Spain).

**Figure 1:**
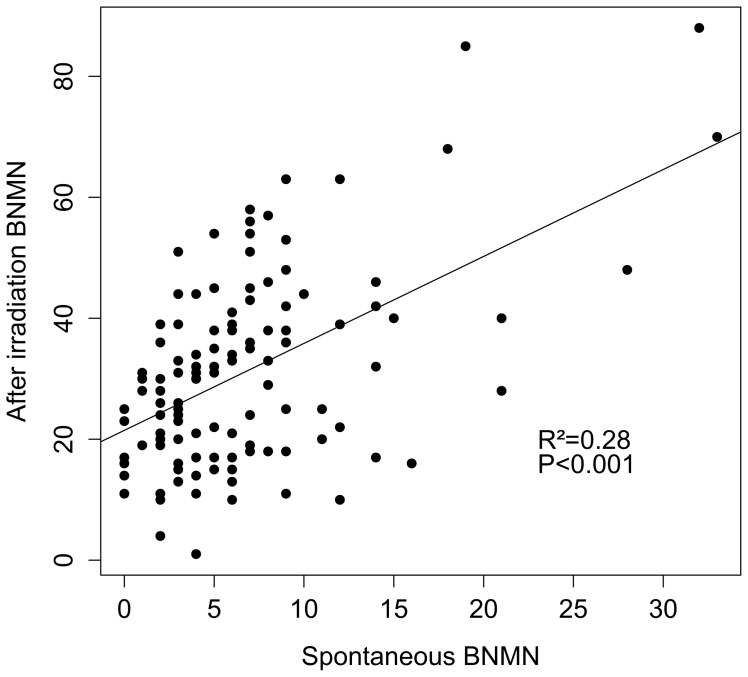
Correlation between DNA damage values (spontaneous *vs* after irradiation).

### Population studied

The study was carried out in a total of 115 thyroid cancer patients, 93 (81%) women and 22 (19%) men, recruited from both the Hospital Universitari Vall d'Hebron in Barcelona, and the Hospital Universitari Doctor Josep Trueta in Girona (Spain). The average age at diagnosis was 43.30±15.07 (mean ± SD) years and 49.76±16.49 when the sample was obtained. According to the tumour-type, 99 (86%) of patients were classified as papillary and 16 (14%) as follicular.

Blood samples were collected prior to iodine treatment (^131^I), after obtaining a written informed consent of all patients. All participants completed a questionnaire, covering standard demographic questions, as well as a brief occupational, medical, and family history. The histological classification of the tumour was obtained from the medical records. All participants signed a written consent.

**Table 2:** Differences for basal DNA damage (BNMN), according to *WDR3* polymorphisms.

	Genotype	N	mean BNMN ± SE	Difference (95% CI)*	*P*
**rs3754127**	**C/C**	39	8.38±1.25	0	0.34
	**C/T**	55	6.07±0.63	−1.77 (−4.13 *to* 0.59)	
	**T/T**	21	6.14±0.97	−0.94 (−4.03 *to* 2.15)	
**rs3765501**	**G/G**	35	8.94±1.36	0	0.28
	**G/A**	43	6.42±0.78	−1.84 (−4.47 *to* 0.78)	
	**A/A**	30	5.70±0.72	−2.12 (−5.04 *to* 0.80)	
**rs4658973**	**T/T**	39	8.38±1.25	0	0.41
	**T/G**	53	6.13±0.65	−1.65 (−4.06 *to* 0.76)	
	**G/G**	21	6.24±0.95	−1.15 (−4.24 *to* 1.95)	

SE: standard error; CI: confidence interval.

* Adjusted by gender, age and diagnosis.

### Cell culture and treatment with IR

The cytokinesis-block micronucleus assay (CBMN) was carried out using the cytochalasin B technique and following a standard procedure [14]. Blood samples were taken by venipuncture and 0.5 mL of heparinised blood was diluted with 4.5 mL of complete culture medium consisting of RPMI 1640, supplemented with 15% foetal bovine serum, 1% antibiotics (penicillin and streptomycin) and 1% l-glutamine; all chemical were obtained from PAA Laboratories GmbH, Pasching, Austria. Lymphocytes were stimulated to divide with 1% phytohaemagglutinin (PHA) (Gibco San Diego, USA) immediately after the treatment with IR.

**Table 3:** Differences for basal DNA damage, according to *WDR3* haplotypes.

	rs3754127	rs3765501	rs4658973	Frequency	Difference (95% CI)	*P*
1	C	G	T	0.5226	0	–
2	T	A	G	0.4217	−0.74 (−2.26 *to* 0.78)	0.34
3	C	A	T	0.0513	−1.96 (−5.15 *to* 1.23)	0.23
rare	*	*	*	0.0043	−5.79 (−16.98 *to* 5.39)	0.31

Global haplotype association P-value: 0.31.

Four cultures were established per each donor. Two were irradiated with 0.5 Gy 137Cs γ-rays, while the other two remain untreated. Irradiation was carried out at the Unitat Tècnica de Protecció Radiològica (UTPR-UAB) of the Universitat Autònoma de Barcelona. The dose rate was 6.00 Gy min^−1^. Lymphocytes (irradiated and non-irradiated) were cultured at 37°C for 72 hours. At 44 hours after PHA stimulation, 10 μL (3 mg/mL) of cytochalasin B (Sigma-Aldrich, St. Louis, USA) was added to give a final concentration in culture of 6 μg/mL. Cultures were maintained at 37°C and 5% CO_2_.

**Table 4:** Differences for after-irradiation DNA damage, according to *WDR3* polymorphisms.

	Genotype	N	mean BNMN ± SE	Difference (95% CI)*	*P*
**rs3754127**	**C/C**	39	37.46±2.91	0	**0.021^C^**
	**C/T**	55	27.87±1.77	**−9.24 (−15.76** *to* **−2.71)**	
	**T/T**	21	29.1±3.55	−7.77 (−16.31 *to* 0.77)	
	**C/C**	39	37.46±2.91	0	**0.0057^D^**
	**C/T-T/T**	76	28.21±1.6	**−8.85 (−15.01** *to* **−2.70)**	
**rs3765501**	**G/G**	35	38.09±3.17	0	**0.034^C^**
	**G/A**	43	29.14±2.07	**−8.77 (−16.03** *to* **−1.51)**	
	**A/A**	30	28.33±2.73	**−9.33 (−17.41** *to* **−1.24)**	
	**G/G**	35	38.09±3.17	0	**0.0091^D^**
	**G/A-A/A**	73	28.81±1.65	**−8.98 (−15.61** *to* **−2.36)**	
**rs4658973**	**T/T**	39	37.46±2.91	0	**0.025^C^**
	**T/G**	53	27.89±1.82	**−9.23 (−15.87** *to* **−2.59)**	
	**G/G**	21	29.71±3.55	−7.30 (−15.84 *to* 1.23)	
	**T/T**	39	37.46±2.91	0	**0.0074^D^**
	**T/G-G/G**	74	28.41±1.64	**−8.70 (−14.94** *to* **−2.46)**	

SE: standard error; CI: confidence interval.

* Adjusted by gender, age and diagnosis.

C, D: Codominant and Dominant models, respectively.

### Cell harvesting and MN scoring

Cells were collected by centrifugation (10 min at 120 g). The supernatant was discarded after each centrifugation. The hypotonic treatment was carried out adding 5 mL of KCl 0.075 M at 4°C for 10 min. Next, cells were centrifuged and a methanol/acetic acid mixture (3∶1 v/v) was gently added. This fixation step was repeated twice and the resulting cells were re-suspended in a small volume of fixative solution. Air-dried preparations were made and the slides were stained with 10% Giemsa (Merck KGaA, Darmstadt, Germany) in phosphate buffer (pH 6.8) for 7 min. Slides were coded by a person does not involved in the scoring.

**Table 5:** Differences for after-irradiation DNA damage, according to *WDR3* haplotypes.

	rs3754127	rs3765501	rs4658973	Frequency	Difference (95% CI)	P-value
**1**	C	G	T	0.5227	0	–
**2**	T	A	G	0.4217	**−4.9 (−9.16** *to* **−0.63)**	**0.026**
**3**	C	A	T	0.0512	−3.74 (−12.73 *to* 5.26)	0.42
**rare**	*	*	*	0.0043	4.21 (−27.15 *to* 35.58)	0.79

Global haplotype association P-value: 0.79.

To detect the level of genetic damage, the frequency of binucleated cells with micronuclei (BNMN) was evaluated. A blinded scoring of 1000 binucleated cells per sample (irradiated and non-irradiated) were analyzed to determine the presence of micronuclei, according to standard criteria [14].

### SNP selection

The polymorphisms used in this study were selected according to our previous analyses [3]. Thus, we selected three tag SNPs showing haplotype association with thyroid cancer, rs3754127 (C/T, 5′ near gene), rs3765501 (G/A, intron) and rs4658973 (T/G, intron).

### DNA extraction and SNP genotyping

DNA was isolated from peripheral blood, using the standard phenol-chloroform method. The SNP genotyping was performed at the Centro Nacional de Genotipado (CeGen) using the iPLEX (Sequenom) technique and it was successfully genotyped according to the quality control criteria of the CeGen (http://www.cegen.org). To assure the genotyping reliability, 10% of the samples were randomly selected and double genotyped by replicates in multiple 96 well plates. In addition, two HapMap reference trios were incorporated in plates and the genotype concordance and correct Mendelian inheritance were verified.

### Statistical analysis

The analysis of differences, linkage disequilibrium and haplotype frequency estimation were performed using the web tool for SNP analysis SNPStats [15]. Linear regression model for dominant and codominant inheritance were performed. All analyses were adjusted for age, gender and histological diagnosis. Haplotype analyses were performed using the same web tool. Haploview software [16] was used to examine the LD between SNPs. Other statistical analysis were done using R version 2.12.1 [17].

## Results

General characteristics of the selected DTC patients are summarized in the Material and Methods section. As indicated, the proportion of women was higher than that of men, reflecting the highest incidence of DTC in women. In addition, the elevated incidence of the papillary thyroid cancer indicates the predominance of this histological subtype.

Table 1 shows that the mean of spontaneous BNMN frequency in the 115 patients was 6.87±0.55 (mean ± SE). The BNMN values showed a 4.6 fold increase (31.35±1.5) after exposure to 0.5 Gy of ionizing radiation, regarding to the spontaneous levels. These differences between the spontaneous and after irradiation BNMN frequency were, as expected, statistically significant (P<0.001). Moreover, although the mean of spontaneous BNMN was higher in females (7.19±0.65) than in males (5.51±0.94) this difference did not attain statistical significance (P = 0.339). On the other hand, no differences were observed between females and males after irradiation (31.19±1.70 and 31.01±3.11; respectively, P = 0.833).

The spontaneous BNMN values in the subtype of patients with papillary thyroid cancer showed a slight but not significant decrease, in comparison with the follicular subtype (6.64±0.57 and 8.31±1.82; respectively, P = 0.559). The values of BNMN after irradiation neither shown significant differences between both subtypes (31.71±1.50 and 29.13±5.53; respectively, P = 0.553).

In Figure 1 it is observed the good correlation that exists between the spontaneous BNMN values and those observed after the irradiation (R^2^ = 0.28, P<0.001). This would indicate an underlying genetic cause.

We have also evaluated the cellular proliferation rate using the cytokinesis-blocked proliferation index (CBPI). The CBPI value indicates the average number of cell cycles per cell [18]. The results (data not shown) indicate a significant decrease of CBPI in cultures exposed to ionizing radiation (P<0.001).

The differences and their 95% confidence intervals were calculated by linear regression analyses using as a reference the homozygous genotype for the most frequent allele, with adjustment for age, gender and diagnoses. Table 2 shows the differences observed on spontaneous BNMN values, according to the different polymorphisms, taking into account the codominant model of inheritance. For the spontaneous BNMN frequency small differences between genotypes were found for all the evaluated polymorphisms. Interestingly, in all the cases the BNMN values were higher in the reference genotypes (homozygous for the common allele), but these differences did not attain statistical significances.

Haplotype analysis was performed for the three SNPs of WDR3 and the results are indicated in Table 3. Three haplotypes were predicted to have a frequency >0.01. The other haplotypes were predicted to be too odd for meaningful statistical analysis. No significant differences were observed for any of the most frequent haplotypes with the spontaneous BNMN values.

The means for the BNMN frequencies were significantly different by genotype among the three *WDR3* polymorphisms (Table 4). We observed significantly lower mean BNMN frequencies in the presence of the minor allele after ionizing radiation exposure suggesting a dominant model of inheritance. For example, the rs3754127 C/T-T/T genotypes showed a decreased BNMN frequency compared with CC individuals (see Table 4). Similarly, we also found a decrease in the radiation-induced damage by haplotype when combining the minor allele of each of the three genotyped SNPs [−4.9 (−9.16 *to* −0.63), P = 0.026] (see Table 5).

When the analysis was performed stratifying the population by the type of tumour at diagnosis (follicular or papillary), the papillary subgroup showed values after irradiation similar to those of the overall population: rs3754127 [−8.39 (−14.45 to −2.33), P<0.01]; rs3765501 [−8.83 (−15.37 to −2.29), P<0.01]; rs4658973 [−8.36 (−14.46 to −2.27), P<0.01], all for the dominant model. On the other hand, the effect observed for the follicular subtype after irradiation was higher than in papillary and in the overall DTC patients; however, the differences were not statistically significant, probably due to low numbers of individuals: rs3754127 [−27.78 (−55.54 to −0.02), P = 0.074]; rs3765501 [−28.67 (−62.46 to 5.12), P = 0.12]; rs4658973 [−29.10 (−60.26 to 2.06), P = 0.094], all for dominant model.

## Discussion

In this study we have shown a significant relationship between the three selected polymorphisms of the *WDR3* gene and the levels of DNA damage after a treatment with IR. This would indicate a role of WDR3 in the maintenance of genomic stability.

*WDR3* gene encodes a nuclear protein containing 10 WD repeats with unknown function [4] and the members of this large family are structurally related, but functionally diverse. Some of the cellular functions or pathways regulated by the members of this family includes gene regulation, signal transduction, RNA processing and splicing, lymphocyte homing, regulation of cell cycle progression, cell division/chromatin separation, and cell/tissue differentiation, among other, as reviewed [5], [6]. However, up to now there is not information indicating a possible involvement with repair mechanisms or influence, in the maintenance of genomic stability for *WDR3*, or for any member of its family.

Aiming to find a reasonable explanation for the association observed between *WDR3* polymorphisms and DTC incidence, we have already reported the up-regulation of *WRD3* in different thyroid cancer cell lines, suggesting its possible implication in thyroid cancer tumorigenesis [3]. This would agree with the studies carried out in the breast carcinoma cell line MCF-7 showing that the suppression of *WDR3* reduce cell proliferation, decrease cell size and reduce foci formation, indicating that *WDR3* confers a growth and proliferative advantage on cancer cells [8]. The WDR3 protein is redistributed within the nucleus when ribosome biogenesis is disrupted. Cellular events appear to be influenced by the regulation of expression of *WDR3* altering ribosome biogenesis [8]. Decreased transient changes on the levels of DNA-damage repair proteins (non-homologous end-joining proteins) have been observed in the nucleolus after ionizing radiation exposure; therefore, it seems possible that these alterations reflect a biologically relevant response to DNA double-strand break damage [19].

Our findings suggest a correlation between the minor frequency alleles for rs3754127, rs3765501, rs4658973 polymorphisms and lower values of DNA damage, after the treatment with ionizing radiation. Although the SNPs used in our study do not produce changes of amino acids in the protein sequence, it is known that non-coding single nucleotide polymorphisms may alter gene expression [20]. Modifications near the promoter sequence (rs3754127) can interrupt the correct interaction with transcription factors changing totally the gene expression or influencing the level of expression [21]; this means that the transcription of WDR3 could depend of genetic variance at its promoter region. Although alternative splicing, due to the presence of SNPs, probably occur [22], [23], it must be pointed out that alternative transcripts are also induced by ionizing radiation [24]. Thus, polymorphisms at rs3765501 and/or rs4658973 may also influence alternative splicing, not only because of its presence but also by additional or differential effects with the ionizing radiation effects. Alternatively, any of these polymorphisms may be responsible for the decrease of DNA damage after IR, but they act as genetic markers of genetic variants of WDR3 directly involved in the WDR3 genomic stability.

The observed effects, indicating the involvement of *WDR3* in maintaining genomic stability after the exposure to IR, would agree with the association found with DTC and with the role attributed in the ribosome biogenesis. Thus, alterations in the expression of *WDR3* would disrupt the signalling pathways that exist between ribosome biogenesis and p53 activation [8]. All these results altogether suggest an important role of *WDR3* in maintaining genome stability as well as in carcinogenesis and cell cycle regulation. Interesting, preliminary results with colon cancer patients (V. Moreno, personal communication) seem to indicate an altered expression of *WDR3* in colon cancer, supporting our view, proposing a role of WDR3 in genomic stability and cancer. Our data are interesting enough to carry out additional studies in *WDR3* gene expression to confirm this hypothesis.
